# Benefits and safety of gabapentinoids in chronic low back pain: A systematic review and meta-analysis of randomized controlled trials

**DOI:** 10.1371/journal.pmed.1002369

**Published:** 2017-08-15

**Authors:** Harsha Shanthanna, Ian Gilron, Manikandan Rajarathinam, Rizq AlAmri, Sriganesh Kamath, Lehana Thabane, Philip J. Devereaux, Mohit Bhandari

**Affiliations:** 1 Department of Anesthesiology, St Joseph’s Healthcare, McMaster University, Hamilton, Ontario, Canada; 2 Department of Health Research Methods, Evidence, and Impact, McMaster University, Hamilton, Ontario, Canada; 3 Departments of Anesthesiology & Perioperative Medicine and Biomedical & Molecular Sciences, Queen's University, Kingston, Ontario, Canada; 4 Department of Neuroanesthesia, National Institute of Mental Health and Neuro Sciences, Bangalore, India; 5 Biostatistics Unit, St Joseph's Healthcare, Hamilton, Ontario, Canada; 6 Department of Medicine, McMaster University, Hamilton, Ontario, Canada; 7 Department of Surgery, McMaster University, Hamilton, Ontario, Canada; Massachusetts General Hospital, UNITED STATES

## Abstract

**Background and objective:**

Chronic Low Back Pain (CLBP) is very common, with a lifetime prevalence between 51% and 80%. In majority, it is nonspecific in nature and multifactorial in etiology. Pregabalin (PG) and Gabapentin (GB) are gabapentinoids that have demonstrated benefit in neuropathic pain conditions. Despite no clear rationale, they are increasingly used for nonspecific CLBP. They necessitate prolonged use and are associated with adverse effects and increased cost. Recent guidelines from the National Health Service (NHS), England, expressed concerns on their off-label use, in addition to the risk of misuse. We aimed to assess the effectiveness and safety of gabapentinoids in adult CLBP patients.

**Methods:**

Electronic databases of MEDLINE, EMBASE, and Cochrane were searched from their inception until December 20^th^, 2016. We included randomized control trials reporting the use of gabapentinoids for the treatment of CLBP of >3 months duration, in adult patients. Study selection and data extraction was performed independently by paired reviewers. Outcomes were guided by Initiative on Methods, Measurement and Pain Assessment in Clinical Trials guidelines, with pain relief and safety as the primary outcomes. Meta-analyses were performed for outcomes reported in 3 or more studies. Outcomes were reported as mean differences (MDs) or risk ratios (RRs) with their corresponding 95% confidence intervals (CIs), and I^2^ in percentage representing the percentage variability in effect estimates that could be explained by heterogeneity. GRADE (Grading of Recommendations Assessment, Development, and Evaluation) was used to assess the quality of evidence.

**Results:**

Out of 1,385 citations, eight studies were included. Based on the interventions and comparators, studies were analyzed in 3 different groups. GB compared with placebo (3 studies, *n* = 185) showed minimal improvement of pain (MD = 0.22 units, 95% CI [−0.5 to 0.07] I^2^ = 0%; GRADE: very low). Three studies compared PG with other types of analgesic medication (*n* = 332) and showed greater improvement in the other analgesic group (MD = 0.42 units, 95% CI [0.20 to 0.64] I^2^ = 0; GRADE: very low). Studies using PG as an adjuvant (*n* = 423) were not pooled due to heterogeneity, but the largest of them showed no benefit of adding PG to tapentadol. There were no deaths or hospitalizations reported. Compared with placebo, the following adverse events were more commonly reported with GB: dizziness-(RR = 1.99, 95% CI [1.17 to 3.37], I^2^ = 49); fatigue (RR = 1.85, 95% CI [1.12 to 3.05], I^2^ = 0); difficulties with mentation (RR = 3.34, 95% CI [1.54 to 7.25], I^2^ = 0); and visual disturbances (RR = 5.72, 95% CI [1.94 to 16.91], I^2^ = 0). The number needed to harm with 95% CI for dizziness, fatigue, difficulties with mentation, and visual disturbances were 7 (4 to 30), 8 (4 to 44), 6 (4 to 15), and 6 (4 to 13) respectively. The GRADE evidence quality was noted to be very low for dizziness and fatigue, low for difficulties with mentation, and moderate for visual disturbances. Functional and emotional improvements were reported by few studies and showed no significant improvements.

**Conclusions and relevance:**

Existing evidence on the use of gabapentinoids in CLBP is limited and demonstrates significant risk of adverse effects without any demonstrated benefit. Given the lack of efficacy, risks, and costs associated, the use of gabapentinoids for CLBP merits caution. There is need for large high-quality trials to more definitively inform this issue.

**Trial registration:**

PROSPERO CRD42016034040

## Abstract 2

In a systematic review and metanalysis, Harsha Shanthanna and colleagues examine the evidence for the benefits and safety of gabapentoids for chronic low back pain.

## Author summary

**Why was this study done?:**

- Chronic low back pain (CLBP) is widely prevalent, and in majority it is nonspecific (no clear etiology) in nature. Among chronic conditions, CLBP is noted to be the leading cause of years lived with disability.
- Gabapentin (GB) and Pregabalin (PG) have been shown to be helpful in neuropathic pain conditions, such as diabetic neuropathy. Despite no clear rationale, their use for CLBP has significantly increased.
- We examined the existing literature and strength of evidence to determine the usefulness of either PG or GB in decreasing pain and improving functions, and the potential adverse effects of PG and GB, in patients with predominant CLBP.

**What did the researchers do and find?:**

- We performed a systematic review and meta-analysis of randomized control studies that used either PG or GB in patients of predominant CLBP.
- We identified only 8 randomized control studies that assessed the benefits of using GB or PG in CLBP.
- While GB showed minimal improvement of pain compared to placebo, pain relief with PG was inferior compared to the active analgesic group. GB and PG were both associated with increased risk of dizziness compared with placebo or active comparator, respectively. GB was additionally associated with increased risk of fatigue, visual disturbances, and difficulties with mentation compared with placebo.

**What do these findings mean?:**

- There is limited evidence to support the use of either PG or GB in nonspecific CLBP.
- The limited and low-quality evidence suggests increased risk of adverse effects with only minimal benefit for GB compared with placebo and no evidence for benefit with PG compared with other analgesics.
- Their continued use in CLBP merits caution.

## Introduction

Chronic Low Back Pain (CLBP) is very common and is associated with significant patient burden and heath resource expenditure [1–3]. It is largely nonspecific in nature and in up to 85% of patients lacks a clear pathoanatomical diagnosis when present in isolation [1–4]. We have previously highlighted the etiological and treatment considerations for CLBP, along with the limitations within the existing evidence [5]. A large proportion of CLBP patients are treated with routine analgesic medications with unsatisfactory results leading to frequent exploration of second line options including gabapentinoids [6, 7]. In particular, the use of gabapentin (GB) and pregabalin (PG) is made on the rationale of modulating the enhanced neurotransmission at the level of presynaptic receptors of the afferent neurons. Both of these medications primarily act on the α-2 delta-2 subunit of the voltage-dependent calcium channels [8, 9] and can be considered to have very similar pharmacodynamic actions on pain and other symptoms. They are considered to be very effective for neuropathic pain (NP) conditions. Attempts at exploiting their therapeutic potential for other pain conditions have shown mixed results [10, 11]. Use of gabapentinoids for CLBP requires slow titration to therapeutic doses and establishing maintenance on a long-term basis. With prolonged treatment, the potential gain over possible adverse effects and risks could become unclear [9]. There have been concerns over the excessive off-label use of GB, despite there being a clear lack of clinical studies [12], necessitating advisory guidelines by the National Health Services (NHS), United Kingdom on the risk of the misuse of gabapentinoids [13]. Our primary objectives were to assess the benefits of GB and PG in CLBP in decreasing pain and to examine the risk of adverse effects. Secondarily, we assessed the effects of PG and GB on the Initiative on Methods, Measurement and Pain Assessment in Clinical Trials (IMMPACT) outcomes [14]. The outcomes considered were physical and emotional functioning, participant ratings of global improvement and satisfaction with treatment, and participant disposition. Additionally, we attempted to assess whether the use of gabapentinoids selectively improve pain relief in patients with predominant neuropathic CLBP.

## Methods

As this is a systematic review, ethics committee approval is not applicable.

### Protocol and registration

Our review was registered with PROSPERO with the registration number CRD42016034040. This report has been prepared according to PRISMA guidelines [15], as suggested by the Enhancing the QUAlity and Transparency Of health Research (EQUATOR) network (S1 PRISMA checklist). Our detailed review protocol has been previously published [5].

### Eligibility criteria

We included randomized controlled trials (RCTs) involving adult patients (>18 years of age) with predominant CLBP of 3 months or more, with or without leg pain. We did not have any language exclusions. Studies with mixed population of chronic pain were only included if they report outcomes separately for our study population of interest, or if at least 90% of the trial patients are >18 years with predominant CLBP. Studies were further screened for interventions and were included if they randomized patients to receive “PG” or “GB,” either “alone” or “in combination with other treatment,” and compared it with any active or inactive treatments.

### Information sources

We searched the electronic databases of EMBASE, MEDLINE, and the Cochrane Central Registry of Controlled Trials (CENTRAL), from their inception until January 26^th^, 2016. WHO clinical trial registry (http://apps.who.int/trialsearch/Default.aspx), and clinical trial registry (https://clinicaltrials.gov/), were also searched to look for any registered studies, fulfilling our eligibility criteria, and crosschecked for their resulting publications. To be comprehensive, bibliographies of relevant reviews and selected studies were examined. Since performing the original search, we also repeated our search on December 20^th^, 2016 to ensure that we have not missed any recent publications.

### Search strategy

The search was performed using a sensitive strategy by an experienced librarian for each specific database. We included terms referring to study population of low back pain, and terms referring to study interventions such as GB, PG, and anticonvulsants [5]. The strategy is provided as a supplementary file (S1 Text).

### Study screening and selection

Using paired reviewers screening independently and in duplicate, study selection was performed in 2 stages. Titles and abstracts were screened in the first stage, followed by full text screening on citations felt potentially eligible. A calibration exercise between reviewer pairs ensured consistency in screening and disagreement were resolved by consensus or through discussion with the principal investigator (HS). A quadratic kappa statistic on the full article final decision was estimated as a measure of interobserver agreement [16].

### Data collection process

The same paired reviewers extracted the data independently and in duplicate, using electronic data extraction forms that were piloted between the reviewers for consistency and accuracy. An instruction manual was provided to assist with the data extraction process.

### Data items

Data items extracted from each study included study characteristics, risk of bias (RoB) items, demographic information, participant disposition through the study, and our review outcomes on continuous and binary measures captured on 6 core domains as recommended by the IMMPACT statement guidelines [14].

### RoB in individual studies

RoB was assessed using the Cochrane RoB tool modified to capture the components of random sequence generation; allocation concealment; blinding of participants; blinding of outcome assessment; and analysis of incomplete outcome data. Further, we modified the response options of domains as “definitely yes,” “probably yes,” “probably no,” and “definitely no.” For each domain, the responses of “definitely yes” and “probably yes” categories were assigned a high RoB and those in the “probably no” and “definitely no” categories a low RoB[17]. Crossover studies were assessed for reasonable washout period [18]. No attempt was made to contact authors for clarification on the RoB items. Selective outcome reporting was judged based on the outcomes described in the methods section but not reported in the results section [19].

### Additional RoB items

Additionally, we considered the domains for chronic pain studies as suggested by Moore et al. [20] and added the domains of outcome assessment time (12 weeks or more as low risk), outcome assessment threshold (>30% improvement in pain relief as low risk), and potential for publication bias based on the sample size threshold (>50 as low risk) to identify a trial as having the potential for publication bias based on low sample size. Trials with low sample size can increase the chances of erroneously large treatment effect sizes and indirectly contribute to publication bias [21, 22].

### Outcomes and prioritization

A priori, we specified pain relief and safety (adverse effects) as our primary outcomes and others as secondary outcomes, and prioritized the use of intent to treat analysis. Pain relief expressed as both continuous and categorical outcomes, and at various time points, was extracted for all reported time points. For pooling, we considered the most common type and the longest duration of follow-up reported. A priori, we prioritized change scores over end scores for pooling analysis. Change scores are considered more efficient and powerful than comparison of final scores, as it removes a component of between-person variability from the analysis [18]. For pain relief expressed as continuous scores, we converted all study outcomes into a common 0–10 numerical rating scale, as it is commonly used and easy to interpret [14]. The approach to conversion into a common scale is shown in S2 Text. Safety was assessed by comparing the risk of serious adverse events causing death, hospitalisation, or study withdrawal. If unclear, we considered reporting the most commonly reported adverse effects. Due to the expected differences within measurement scales, secondary outcomes of improvement in physical and emotional functioning, and participant ratings of global improvement and satisfaction were not converted into a single common scale.

### Synthesis of results and summary measures

Data were pooled only if there are 3 or more studies contributing to an outcome domain. Our selection criteria allowed for a relatively homogeneous population of CLBP who tend to be approached similarly from a clinical situation. However, we recognized the potential for heterogeneity based on study interventions and comparator interventions. In view of these obvious sources of heterogeneity, we decided a priori to pool studies using PG or GB, either alone or in combination, separately. Extracted data were compiled and checked for accuracy using Microsoft Excel. RoB was assessed using a modified Cochrane RoB tool that is described below. For the primary analysis, we used a complete case analysis, as reported in individual studies. Sensitivity analyses for incomplete outcome data were performed. Analysis and synthesis was carried out using Review Manager (RevMan) [Computer program], Version 5.3. Copenhagen: The Nordic Cochrane Centre, The Cochrane Collaboration, 2014; and Microsoft Excel 2011 (Mac version). Based on the comparator and interventions, if we did not expect much between study variance, a fixed effects model was used for pooling. However, if we suspected between study variance, or in the presence of unexplained heterogeneity, a random effects model was chosen [18]. For crossover studies, we prioritized the results from a paired test. If not provided, results of unpaired tests were considered. If there was a potential for carryover effect, or if there is a significant drop out rate (>20%), the results from the first period only were considered [18]. Statistical heterogeneity was estimated using Cochrane’s Q test, with a threshold of *p*-value at 0.10, and the percentage variability in individual effect estimates was described by I^2^ statistic [18]. Risk Ratio (RR), and mean difference (MD) or standardized mean differences (SMDs) as appropriate, were estimated along with their 95% confidence intervals (CI). We planned to report the findings in measures of absolute risk, if they were observed to be statistically significant. Rating of quality of evidence was done using GRADE approach, with a summary of findings (SOF) table.

### Additional analysis

A subgroup analysis was considered in studies that screened for the presence of NP using a screening questionnaire at baseline and reported pain relief in patients of NP separately. Sensitivity analyses for the outcome of pain relief was carried out for studies reporting >5% loss to follow-up (LTFU). These were carried out using well-described imputation strategies [23, 24].

## Results

### Study selection

**Fig 1:**
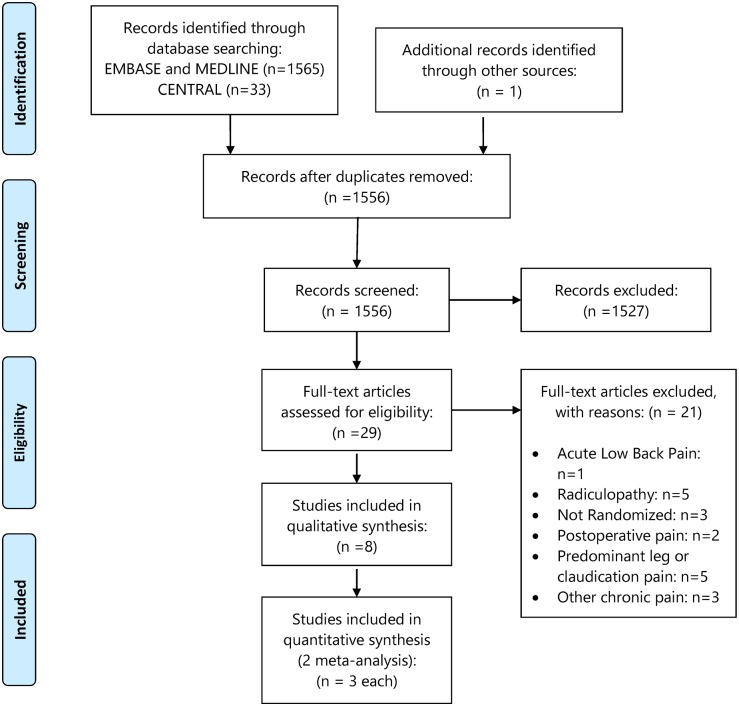
PRISMA flow diagram.

Our search identified a total of 1,385 citations after exclusion of duplicates. Among the 29 articles assessed for full text, 21 studies were excluded with reasons that are shown in Fig 1. Eight studies were included for qualitative and six for quantitative analysis (Fig 1). There was almost perfect agreement, indicated by kappa = 0.82, between reviewers at the full-text screening stage.

### Study characteristics

Important characteristics of the study population and treatments are provided in Table 1. Of the 8 studies, 3 compared the use of GB to placebo treatment [25, 27], and 5 used PG [28–32]. There were 2 crossover studies using GB [26] and PG [31]. Only 2 studies were multicentered and had external funding for the conduct of their trial [25, 28]. Among the PG trials, 3 trials used an active comparator (amitryptline, celebrex, tramacet) versus PG alone [29, 31, 32]. As the study by Romano et al. had 3 arms [31], they compared PG alone versus celebrex (CX) versus a combination of PG plus CX. So, there were 3 comparisons involving PG as an adjunct to an analgesic medication versus their respective analgesic medication [28, 30, 31]. The mean age ranged between 41.6 to 58.5 years, except in the study by Sakai et al. [32]. However, the duration of pre-existing CLBP had a much wider range of 13 to 213 months. The treatment doses were titrated for clinical effect in all studies, except for Sakai et al., who had a fixed dosing of PG [32]. The doses ranged from 300 to 3,600 mg/day with GB and 100 to 600 mg/day with PG, in divided doses. Only 3 studies assessed specifically for NP using a screening questionnaire [28, 31, 32].

**Table 1:** Characteristics of included studies: design, population, and interventions.

STUDY POPULATION DESIGN AND GROUPS	FEMALES (%)		MEAN AGE (SD)		MEAN DURATION IN MONTHS (SD)		STUDY TREATMENTS		TREATMENT DURATION	PRE-RANDOMIZATION PERIOD & REASON
*Author year; population and design*	INT	CNT	INT	CNT	INT	CNT	INT	CNT		
*Baron 2015* *CLBP >3 months* *2 groups parallel design*	86 (54)	95 (62)	56.3 (11.83)	58.5 (11.01)	104.4 (111.36)	112.8 (125.76)	TAP 300 mg/day + PG 100–200 mg/day	TAP 300 mg/day + TAP 100–200 mg/day	8 weeks	Yes Washout
*Pota 2012*# *CLBP >12 months* *2 groups parallel design*	22/44 (50) in total		55.5 (8.31)		15.25 (8.69)		PG 300 mg/day + BUP 35 mcg/h	BUP 35 mcg/h	3 weeks	Yes to stabilize on BUP for 3 weeks
*Sakai 2015* *CLBP > 3 months* *2 groups parallel design*	9 (30)	11 (37)	72.03 (6.23)	72.60 (5.23)	34.77 (29.91)	34.70 (32.54)	PG 75 mg BID	TRA 2 tablets/day	4 weeks	Yes to washout and rule out acute pain
*Kalita 2014* # *CLBP >3 months* *2 groups parallel design*	91/200 (45.5) in total		42.6 (11.6)	41.6 (10.7)	35.9 (46.8)	35.2 (39.8)	PG 75 mg BID X 2 weeks; 150 mg BID X 4 weeks; 300 mg BID 6–14 weeks	AMT 12.5 mg OD X 2 weeks; 25 mg OD X 4 weeks; 50 mg OD 6–14 weeks	14 weeks	Yes to wash out and treat with NSAIDS If required
*Romano 2009* *CLBP> 6 months* *3 groups; crossover design with 1 week washout; minimal risk of carryover effects*	20 (56)		53 (16)		13 (6)		PG 1mg/kg 1st week; and 2–4 mg/kg next 4 weeks	CX: 3–6 mg/kg	4 weeks	Yes Washout
								PG + CX as with the 2 groups		
*McCleane 2001* *Chronic-duration not provided* *2 groups parallel design*	15 (48)	21 (62)	41.3 (13.1)	47.8 (11.7)	63.1 (45.3)	74.5 (82)	GB 300 mg OD increased weekly to 1,200 mg per day	Similar (placebo capsules)	8 weeks	Yes Not provided
*McCleane 2000* *CLBP >3 months (nociceptive pain);* *crossover design with 1 week washout; minimal risk carryover*	13 (54.2)		42.4 (14.6)		105.5 (97.2)		GB 300 mg daily increasing by 300 mg weekly to a maximum dose of 15 mg/kg	Crossover placebo	6 weeks	No NA
*Atkinson 2016* *CLBP >6 months* *2 groups parallel design with non-inferiority assumption*	12 (18.9)	13 (24.5)	57.58 (8.84)	54.62 (11.38)	205.92 (181.44)	213.48 (153.6)	GB starting as 300 mg/day up to 1,200 mg TID at 4 weeks	Similar (placebo capsules)	12 weeks	No NA

AMT, Amitryptline; BID, twice a day; BUP, Buprenorphine; CLBP, chronic low back pain; CNT, control; CX, Celebrex; GB, Gabapentin; INT, intervention; NSAIDS, Nonsteroidal anti-inflammatory drugs; OD, once a day; PG, Pregabalin; PLA, Placebo; TAP, Tapentadol; TID, three times a day; TRA, Tramacet (37.5 mg Tramadol + 325 mg Acetaminophen); SD, Standard deviation

^#^: Study did not report separately for intervention and control groups

### RoB within studies (Fig 2)

**Fig 2:**
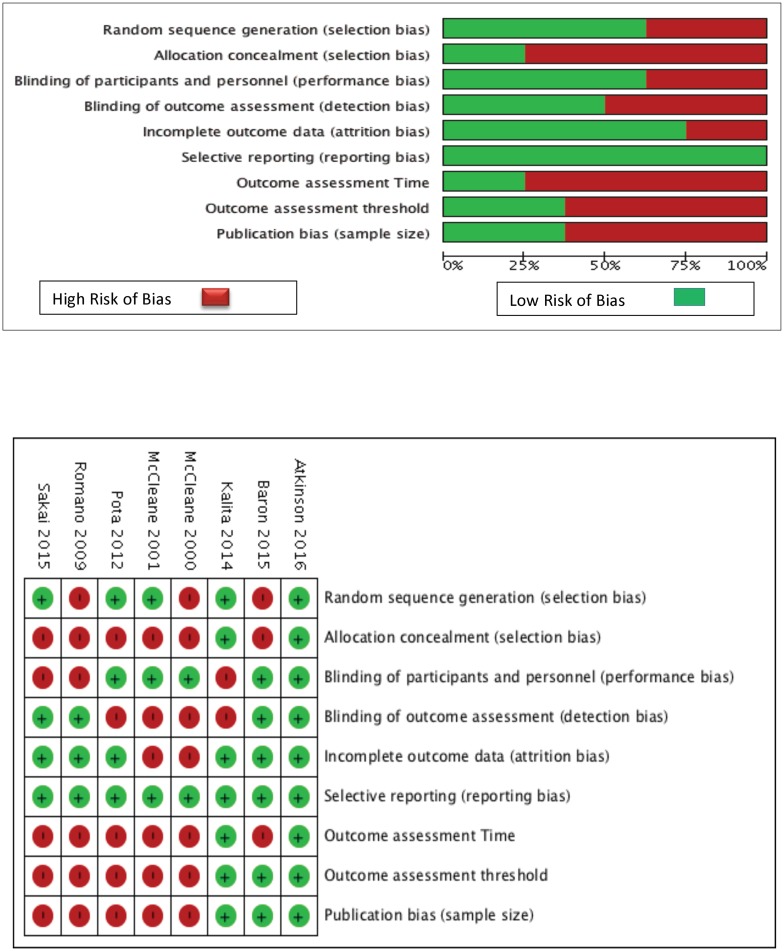
RoB within the included studies. RoB, risk of bias.

Six of the eight studies had a risk of selection bias, six for allocation concealment and three for sequence generation, and four involved a risk of detection bias. The studies by Baron et al. [28], and Atkinson et al. [25] were rated as having low RoB for most domains, and both crossover studies had a higher risk of selection bias [26, 31].

### Study outcomes and synthesis of results

Except 2 studies that reported using 0–100 scale [30, 31], all others reported their pain scores on a scale of 0–10 NRS or Visual Analogue Scale (VAS). Five studies provided a dichotomous measure of treatment success by varying thresholds [25–29, 32]. All studies reported on one or more adverse effects. Functional improvement was reported in 5 studies [25, 26, 28, 29, 32], quality of life (QOL) improvement by 2 studies [28, 32], psychological improvement or improvement in depression by 3 studies [25, 28, 32], and global impression of change (GIC) only by 2 studies [25, 28].

#### Pain relief

**Fig 3:**
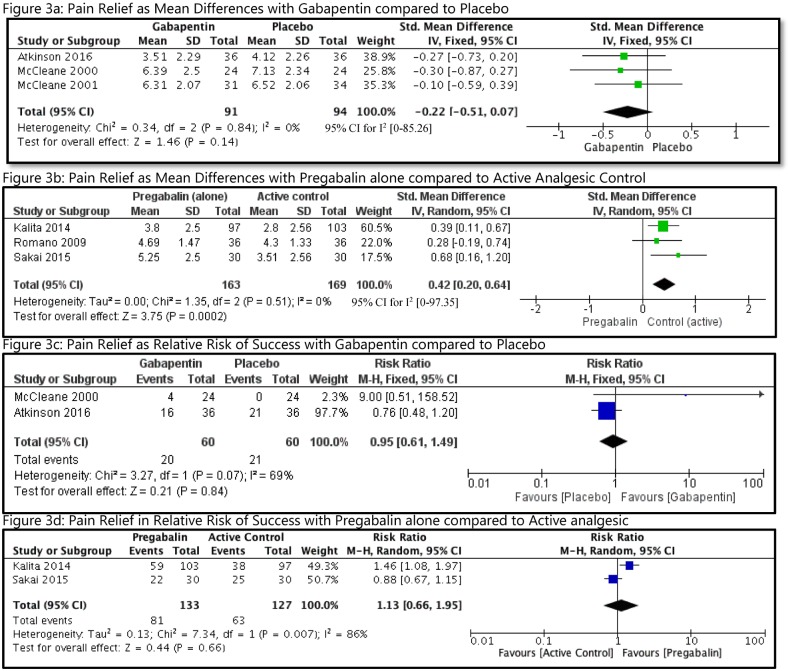
Analyses of pain relief with GB or PG in patients with CLBP. CLBP, chronic low back pain; GB, gabapentin; IV, intravenous; M-H, Mantel-Haenszel; PG, pregabalin.

Pain relief expressed in NRS or VAS scales were converted into a common scale of 0–10 NRS. Authors of 2 studies were successfully contacted to obtain final results of pain scores, as it was not clear in their reporting [25, 32]. We were unable to use the change scores as many studies did not report their change in standard deviations (SDs), and imputing them based on another study or by using a correlation coefficient of change was observed to be inappropriate and not precise [18]. So, pooling was performed using end scores. Based on the variability in the study comparisons, we decided to pool studies for the use of GB and PG. In the first group (Fig 3a), studies using GB (*n* = 91) versus placebo (*n* = 94) were combined using a fixed effects model. Compared with placebo, the GB group had a small reduction in pain (MD = 0.22 units, 95% CI [−0.51 to 0.07], I^2^ = 0%). There were no studies comparing PG with placebo. PG (*n* = 163) was compared with an active comparator (*n* = 169) in 3 studies (Fig 3b), using random effects model. This analysis showed an improvement in pain favoring the use of the active comparator group (MD = 0.42 units, 95% CI [0.20 to 0.64], I^2^ = 0). Both the above comparisons were rated as very low quality evidence by GRADE (Table 2). The third group consisted of comparisons that used PG as an adjunct to another analgesic medication (*n* = 215), such as buprenorphine (BUP) [30], tapentadol (TAP) [28], and CX [30], and compared it with the use of analgesic medication alone (*n* = 208). We decided that it was not appropriate to pool these studies considering the clinical heterogeneity involved within the studies, on the sides of both intervention and comparator. This was supported by the substantial statistical heterogeneity observed with such an attempt using random effects model, I^2^ = 77%. The forest plot for this comparison is shown as S1 Fig. Among these 3 studies, the largest study by Baron et al. did not find any difference by adding PG to TP at their 10-week follow-up [28]. However, the smaller studies by Pota et al. [30] and Romano et al. [31] observed important differences in pain scores (difference of more than 2 points in 0–10 NRS) by using PG as an adjunct to BP and CX, respectively. There were also no significant differences when patients were assessed as success or failure with either GB versus placebo (Fig 3c) or PG versus active comparator (Fig 3d).

**Table 2:** GRADE summary of findings. Gabapentin or pregabalin compared to placebo or active medications for chronic low back pain: A systematic review and meta-analysis of randomized control trials.

Outcomes	№ of participants (studies) Follow-up	Quality of the evidence (GRADE)	Relative effect (95% CI)	Anticipated absolute effects	
				Risk with Placebo or Active medications	Risk difference with Gabapentin or Pregabalin *
Gabapentin compared to Placebo (Pain Relief achieved) assessed with: Patient reported Scale from: 0 to 10 follow up: range 8 weeks to 12 weeks	185 (3 RCTs)	⨁◯◯◯ VERY LOW ^a^^,^^b^^,^^c^	-	-	SMD **0.22 lower** (0.51 lower to 0.07 higher)
Pregabalin alone compared to Active control (Pain Relief achieved) assessed with: Patient reported Scale from: 0 to 10 follow up: range 4 weeks to 14 weeks	332 (3 RCTs)	⨁◯◯◯ VERY LOW ^a^^,^^b^^,^^c^^,^^d^	-	-	SMD **0.42 SD higher** (0.2 higher to 0.64 higher)
Dizziness or Unsteadiness with Gabapentin compared to Placebo assessed with: Patient reported follow up: range 6 weeks to 12 weeks	221 (3 RCTs)	⨁◯◯◯ VERY LOW ^a^^,^^b^^,^^c^	**RR 1.99** (1.17 to 3.37)	225 per 1,000	**223 more per 1,000** (38 more to 534 more)
Fatigue or Lethargy with Gabapentin compared to Placebo (Fatigue) assessed with: Patient reported follow up: range 6 weeks to 12 weeks	221 (3 RCTs)	⨁◯◯◯ VERY LOW ^a^^,^^b^^,^^c^	**RR 1.85** (1.12 to 3.05)	261 per 1,000	**222 more per 1,000** (31 more to 536 more)
Visual disturbances with Gabapentin compared to Placebo (Blurring of vision) assessed with: Patient reported follow up: range 6 weeks to 12 weeks	221 (3 RCTs)	⨁⨁⨁◯ MODERATE ^a^^,^^c^	**RR 5.72** (1.94 to 16.91)	180 per 1,000	**850 more per 1,000** (169 more to 2,867 more)
Dizziness or Unsteadiness with Pregabalin alone compared to Active Control assessed with: Patient reported follow up: range 4 weeks to 14 weeks	332 (3 RCTs)	⨁◯◯◯ VERY LOW ^a^^,^^c^^,^^e^	**RR 2.70** (1.25 to 5.83)	130 per 1,000	**221 more per 1,000** (33 more to 629 more)
Difficulty with Mentation with Gabapentin compared to Placebo assessed with: Patient reported follow up: range 6 weeks to 12 weeks	220 (3 RCTs)	⨁⨁◯◯ LOW ^a^^,^^c^	**RR 3.34** (1.54 to 7.25)	209 per 1,000	**489 more per 1,000** (113 more to 1,307 more)
GRADE Working Group grades of evidence High quality: We are very confident that the true effect lies close to that of the estimate of the effect Moderate quality: We are moderately confident in the effect estimate: The true effect is likely to be close to the estimate of the effect, but there is a possibility that it is substantially different Low quality: Our confidence in the effect estimate is limited: The true effect may be substantially different from the estimate of the effect Very low quality: We have very little confidence in the effect estimate: The true effect is likely to be substantially different from the estimate of effect					

Bibliography: Shanthanna H, Gilron I, Thabane L, Devereaux PJ, Bhandari M, AlAmri R, et al. Gabapentinoids for chronic low back pain: a protocol for systematic review and meta-analysis of randomised controlled trials. BMJ open. 2016;6(11)

CI, Confidence interval; GRADE, Grading of Recommendations Assessment, Development, and Evaluation; RCT, randomized control trial; RR, Risk ratio; SMD, Standardized mean difference

Explanations

^a.^ Studies had risk of selection bias

^b.^ Less than optimal information size

^c.^ Based on low sample size

^d.^ Variations in analgesic treatment and intervention treatment dosages

^e.^ Variations within the control agents used

*The risk in the intervention group (and its 95% CI) is based on the assumed risk in the comparison group and the relative effect of the intervention (and its 95% CI).

#### Adverse effects

**Fig 4:**
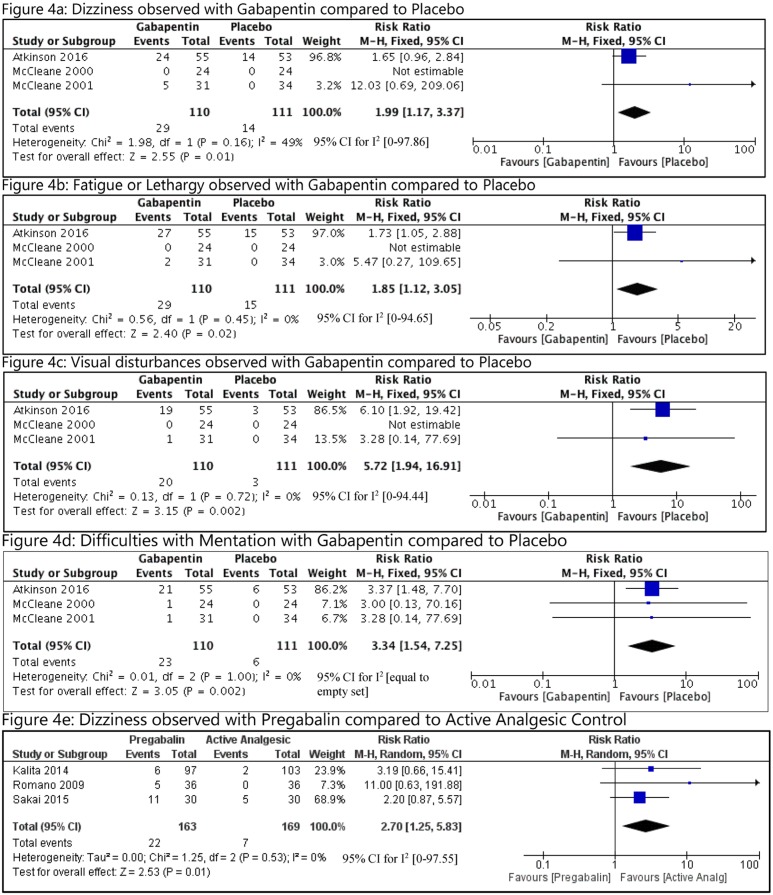
Analyses of adverse effects observed with GB or PG in CLBP. CLBP, chronic low back pain; GB, gabapentin; IV, intravenous; M-H, Mantel-Haenszel; PG, pregabalin.

There were no deaths or hospitalizations reported. The reasons for study withdrawal were not provided in all studies. All adverse effects reported in more than 1 study are summarized in Table 3. Compared with placebo, the following adverse events were more commonly reported with GB: dizziness-(RR = 1.99, 95% CI [1.17 to 3.37], I^2^ = 49); fatigue (RR = 1.85, 95% CI [1.12 to 3.05], I^2^ = 0); difficulties with mentation (RR = 3.34, 95% CI [1.54 to 7.25], I^2^ = 0); and visual disturbances (RR = 5.72, 95% CI [1.94 to 16.91], I^2^ = 0) (Fig 4). The GRADE quality of evidence was noted to be very low for dizziness and fatigue, low for difficulties with mentation, and moderate for visual disturbances (Table 2). The resulting absolute risk increase (ARI) percentage and necessary number needed to harm (NNH) with 95% CI for dizziness, fatigue, mental difficulties, and visual disturbances were 14% and 7 (4 to 30), 13% and 8 (4 to 44), 16% and 6 (4 to 15), and 15% and 6 (4 to 13), respectively. With PG, dizziness was more common compared to the active comparator (RR = 2.70, 95% CI [1.25 to 5.83], I^2^ = 0), with very low quality of evidence. The ARI% and NNH were 9% and 11(6 to 30).

**Table 3:** Summary of adverse effects observed in more than one study.

*Adverse Effects as Described*	BARON 2015		POTA 2012		SAKAI 2015		KALITA 2014		ROMANO 2009		MCCLEANE 2001		MCCLEANE 2000		ATKINSON 2016	
	INT (154)	CNT (159)	INT (22)	CNT (22)	INT (30)	CNT (30)	INT (97)	CNT (103)	INT (36)	CNT (36)	INT (31)	CNT (34)	INT (24)	CNT (24)	INT (55)	CNT (53)
***Nausea/ Vomiting***	20	25	3	3	0	10			PG: 5	CX: 4	6	5	2	2		
										CX + PG: 7						
***Drowsiness/ Somnolence/ Sedation***	19	13	4	5	11	5	4	10			2	0	2	0		
***Forgetfulness/ Memory disturbance***													1	0	9	1
***Constipation***	8	11	5	3	0	6					0	1	1	0	7	9
***Dizziness/Staggering/ Unsteadiness/Vertigo***	28	17	0	22	11	5	6	2	PG: 5	CX: 0	5	0			24	14
										CX + PG: 7						
***Fatigue/Loss of Energy***	16	13									2	0			27	15
***Difficulties with Mentation (Loss of Concentration/ Disorientation/feeling high)***											1	0	1	0	21	6
***Dry Mouth***	8	6					1	3								
***Headache***	13	10									1	1	2	0		
***Problems with Visual Accommodation/ Blurred Vision***											1	0			19	3
***Skin Rash***							1	0			0	1				
***Restlessness***							1	0			1	0				

CNT, control; CX, Celebrex; INT, intervention; PG, Pregabalin

#### Secondary outcomes

These are summarized in Tables 4 and 5. All studies except Pota et al. had patients who were LTFU [30]. There were 5 studies that did include LTFU, even with >5% of their randomized sample, in their final analysis [25–27, 31, 32]. *Functional improvement* was observed in 5 studies using various scales [25, 26, 28, 29, 32]. The results indicate that there were improvements from the baseline in both treatment and control groups, without much difference between the groups. *Emotional functioning* was observed by 3 studies, but 2 studies reported the final scores, with no between-group differences [25, 28]. *Global improvement of change* was reported as physician-reported by Atkinson et al. [25] and patient-reported by Baron et al. [28]. There were no between-group differences in studies with GB or PG, respectively.

**Table 4:** Summary of secondary outcomes-participant disposition.

*PARTICIPANT DISPOSITION*										
*STUDY/YEAR*	RANDOMIZED		COMPLETED STUDY FOLLOW UP		TOTAL LTFU (including withdrawal due to side effects)		LTFU (discontinued study for side effects only)		ANALYZED	
	INT	CNT	INT	CNT	INT	CNT	INT	CNT	INT	CNT
***Baron 2015***	159	154	133	126	26	28	17	16	157#	152#
***Pota 2012***	22	22	22	22	0	0	0	0	22	22
***Sakai 2015***	32	33	2	3	2	3	2	3	30	30
***Kalita 2014***	97	103	70	77	27	26	12	11	97#	103#
***Romano 2009***^a^	42 in each treatment period		36 in each treatment period		6 in each treatment period		4 in each treatment period		36 in each treatment period	
***McCleane 2001***	40	40	31	34	9	6	Not provided		31	34
***McCleane 2000***^b^	30 in each treatment period		24 in each treatment period		6 in each treatment period		1	0	24 in each treatment period	
***Atkinson 2016***	55	53	36	36	19	17	12	6	36	36

CNT, Control; INT, Intervention; LTFU, Loss to follow-up

^a^= triple arm crossover study;

^b^=crossover study;

^#^=performed intent to treat analysis by imputing for patients lost to follow up.

**Table 5:** Summary of secondary outcomes.

***PHYSICIAL FUNCTIONING***						
***STUDY***	**SCALE USED**	**DIMENSION**	**BASELINE**		**END OF STUDY**	
***AUTHOR/ YEAR***			INT	CNT	INT	CNT
***Baron 2015c*** ***INT (159) CNT (154)***	SF-12 physical function composite	0–100 (higher is better)	33.9 (8.49)	34.2 (9.26)	39.6 (9.03)	40.1 (9.64)
***McCleane 2000b*** ***INT (24) CNT (24)***	NRS (mobility scale)	0–10 (higher is better)	4.65 (2.03)	5.07 (2.08)	5.46 (2.41)	5.05 (2.04)
***Atkinson 2016*** ***INT (55) CNT (53)***	ODI	0–100 (lower is better)	40.3 (10.4)	41.1 (9.8)	31.1 (10.6)	30.9 (13.3)
***Sakai 2015*** ***INT (30) CNT (30)***	RDQ	0–24 (lower is better)	9.73 (4.44)	11.47 (4.99)	Not provided as per the treatment and control group	
***Kalita 2014*** ***INT (97) CNT (103)***	ODI	0–100 (lower is better)	42.2 (15.2)	42.2 (12.5)	22 (15)	19 (12.5)
***QOL***						
***STUDY***	**SCALE USED**	**LOWEST TO HIGHEST**	**BASELINE**		**END OF STUDY**	
***AUTHOR/ YEAR***			INT	CNT	INT	CNT
***Baron 2015c*** ***INT (159) CNT (154)***	EQ-5D	0–1 (higher is better)	0.51 (0.246)	0.54 (0.262)	0.60 (0.283)	0.61 (0.305)
***Sakai 2015*** ***INT (30) CNT (30)***	EQ-5D	0–1 (higher is better)	0.63 (0.10)	0.58 (0.12)	Not provided as per the treatment and control group	
***EMOTIONAL FUNCTIONING***						
***STUDY***	**SCALE USED**	**DIMESNSION**	**BASELINE**		**END OF STUDY**	
***AUTHOR/ YEAR***			INT	CNT	INT	CNT
***Baron 2015c*** ***INT (159) CNT (154)***	SF-12 mental health composite	0–100 (higher is better)	47.6 (11.85)	48.8 (11.81)	50 (11.44)	48.2 (10.71)
***Atkinson 2016*** ***INT (55) CNT (53)***	Beck Depression Inventory	0–63 (lower is better)	8.38 (4.32)	8.67 (4.16)	5.79 (3.14)	7.11 (4.60)
***Sakai 2015*** ***INT (30) CNT (30)***	GDI	0–15 (lower is better)	4.70 (3.44)	5.73 (4.25)	Not provided as per the treatment and control group	
***GIC***						
***STUDY***	**SCALE USED**	**CRITERIA**	**END OF TREATMENT FOLLOW UP**			
***AUTHOR/ YEAR***			INT	CNT		
***Baron 2015c*** ***INT (159) CNT (154)***	GIC-patient observed	Minimally improved to very improved	130/157	126/152		
***Atkinson 2016*** ***INT (55) CNT (53)***	GIC-physician observed	Minimally improved to very improved	14/38	11/33		
***NEUROPATHIC PAIN***						
***STUDY/YEAR***	**METHOD OF SCREENING AND NEUROPATHIC PAIN TOOL USED**		**BASELINE**		**END OF TREATMENT/FOLLOW UP**	
			INT	CNT	INT	CNT
***Baron 2015***	Pain DETECT (0–38)		Not reported	Not reported	Decreased by: −6.1 (7.42)	Decreased by: −5.8 (8.66)
***Baron 2015***	NPSI: all patients reported their scores for its individual domains	Overall score (0–100)	46 (18.39)	45.6 (18.52)	29.9 (22.24)	29.8 (22.18)
		Burning pain (0–10)	5 (2.38)	4.7 (2.6)	2.8 (2.69)	3 (2.67)
		Pressing pain (0–10)	4.5 (2.56)	4.6 (2.49)	3.1 (2.52)	3.2 (2.54)
		Paroxysmal pain (0–10)	4.9 (2.29)	4.9 (2.28)	3.3 (2.66)	2.9 (2.53)
		Evoked pain (0–10)	4.2 (2.22)	4.2 (2.28)	2.6 (2.37)	2.6 (2.42)
		Paresthesia/ dysthesia (0–10)	4.8 (2.46)	4.7 (2.61)	3.3 (2.66)	3.4 (2.56)
***Sakai 2015***	NP screening by a Japanese tool with a threshold of >6 as NP+; reported as VAS 0–10 pain scores (INT:13/30; CNT:9/30)		4.56 (3.19)	4.53 (4.46)	6.25	3.43
***Romano 2009***	LANSS with a threshold of >12 as NP+; 16 in each group (crossover study); After 4 weeks of treatment the pain scores in each group were reported (0–100 VAS)		PG: 47.2 (15)	CX: 46.8 (13.6)	PG: 36.3 (12.7)	CX: 45.7 (14.3)
				CX + PG: 47.9 (15.2)		CX + PG: 23.1 (14.6)

CNT, Control; CX, celebrex; EQ-5D, EuroQol 5D; GDI, geriatric depression scale; GIC, global improvement of change; INT, Intervention; LANSS, Leeds assessment of neuropathic symptoms and signs; NP, neuropathic pain; NPSI, neuropathic pain symptom inventory; NRS, numerical rating scale; ODI, Oswestry disability index; PG, Pregabalin; QOL, quality of life; RDQ, Roland Morris questionnaire; SF-12, short form health survey-12; VAS, visual analogue scale.

a=triple arm crossover study;

b=crossover study;

c=baseline scores indicate scores at randomization and not study recruitment

### RoB across studies (Fig 2)

Based on our criteria, potential bias due to outcome threshold, assessment time point, and publication bias due to low sample size was observed largely by 5 studies [26, 27, 30–32].

#### Subgroup analysis

NP was assessed using a screening questionnaire in 3 studies. Sakai et al. observed pain scores to decrease more with tramacet compared to PG in NP patients [32]. Baron et al. observed no differences in the components of neuropathic pain symptom inventory scores using PG plus TP in comparison to TP alone [28]. Whereas, Romano et al. observed that pain scores decreased significantly in patients of NP with PG as well as in combination with CX [31].

#### Sensitivity analysis

The analyses for GB versus placebo, and PG versus active comparator withstood sensitivity analysis for LTFU >5% using progressively stringent imputation strategies for mean pain scores.

## Discussion

Despite the widespread use, our systematic review with meta-analysis found that there are very few RCTs that have attempted to assess the benefit of using GB or PG in patients of CLBP. Use of GB and PG, compared to placebo and active analgesic comparators, respectively, were associated with significant increase in adverse effects without limited evidence for improvement in pain scores or other outcomes. We were unable to examine the pooled effect of using PG as an adjuvant analgesic medication given the limited evidence and heterogeneity of studies. It is reasonable to assume that the clinical benefit would depend upon the primary medication and its potency within each study. The differences within the results of Pota et al. [30] and Romano et al. [31], compared to Baron et al. [28] could be attributed to methodological differences. The study by Baron et al. had a larger sample size along with longer duration of follow up. Hence, the existing evidence does not support the use of gabapentinoids for predominant CLBP, and calls for larger, high quality RCTs to more definitively inform this issue.

Considering the expanding use of gabapentinoids for chronic pain and CLBP [33, 34], this review fulfils the immediate need to scrutinize and closely examine the existing evidence. Noting that there is a published Cochrane protocol [35], ours is the first review combined with meta-analysis to examine the benefits and safety of gabapentinoids in CLBP. Results of our review are in contrast with nonrandomized studies that have shown benefit with PG in patients of CLBP [36, 37]. Gabapentinoids have proven efficacy in NP conditions [38]. However, they are also widely used for conditions in which the neuropathic component is difficult to establish, most of which are off label uses [12]. This development perhaps reflects the penumbra sort of effect (clinicians generalizing the selection criteria of clinical studies into their patient population without recognizing the limitations) [39]. In England, there was a 46% and 53% rise in the prescription use of GB and PG respectively from 2011 to 2013 alone [13]. A recent Canadian study showed that the off-label use of PG is as high as 75%, and the most prevalent condition of use was CLBP [40]. The true burden of NP in CLBP is hard to establish [41]. Distribution of pain can be considered as a corollary of the pathological process, and it is important to broadly classify patients based on their predominance of axial or leg pain for diagnosis and management [2]. A common assumption is of leg pain indicating NP. However, in most cases leg pain is nonspecific and inconsistent with radicular pain, and only a painful radiculopathy with sensory signs would fulfill the diagnosis of definite NP [41]. Even if one considers that gabapentinoids are effective against NP related to CLBP, contrasting evidences are observed in literature. In patients of radicular pain or pain of spinal stenosis, observational studies of CLBP demonstrate significant improvements with PG [42, 43]. However, RCTs performed by Baron et al. in patients of lumbar radiculopathy and Markman et al. in patients of spinal stenosis did not find clinical improvements when PG was compared with placebo [44, 45]. Cohen et al. examined the benefit of GB in patients of leg pain and found no difference as compared to epidural steroid injections [46]. Even within the included study by Baron et al., the reduction of pain and NP symptoms was similar with the combination of PG with TP, compared to TP [28]. Our results are important for practitioners across several specialties who treat patients with CLBP and have to decide on the relative merits and demerits of treatment with gabapentinoids.

Our review is not without its limitations. We excluded studies in patients of predominant leg pain or spinal stenosis. This was done to limit the heterogeneity within our study population. Although the measure of heterogeneity (I^2^-proportion of variability that can be explained by individual studies) was low in many comparisons, the CIs around those I^2^ were very wide, reflecting that there is uncertainty in any claim of homogeneity. Heterogeneity has been shown to be an issue with meta-analyses involving a smaller number of trials or events [47]. Topiramate was not considered in this review, as it has a slightly different mechanism of action and is not commonly used, although some controlled studies have shown benefit [48]. The use of PG or GB is associated with significant adverse effects, cost [13], and potential for misuse [34, 49].

Our review demonstrates that there is limited evidence on the use of gabapentinoids in nonspecific CLBP, and the existing evidence in the form of RCTs does not support their use. It is possible that ongoing or unpublished studies [50, 51] may more definitively inform us on this issue, although one such study specific to CLBP was withdrawn prior to enrollment [52].

## Supporting information

S1 Text Search strategy for MEDLINE and EMBASE. (DOCX)

S2 Text Rescaling or conversion of pain scores to a common 0–10 numerical rating scale. (DOCX)

S1 Fig Forest plot showing comparison of studies using pregabalin as an adjunct analgesic compared to active analgesic. (TIF)

S1 PRISMA checklist (DOC)

## Abbreviations

ARI: absolute risk increase
BUP: buprenorphine
CENTRAL: Cochrane Central Registry of Controlled Trials
CI: confidence interval
CLBP: chronic low back pain
CX: Celebrex
EQUATOR: Enhancing the QUAlity and Transparency Of health Research
GB: gabapentin
GIC: global impression of change
GRADE: Grading of Recommendations Assessment, Development, and Evaluation
IMMPACT: Initiative on Methods, Measurement and Pain Assessment in Clinical Trials
LTFU: loss to follow-up
MD: mean difference
NHS: National Health Service
NNH: number needed to harm
NP: neuropathic pain
PG: pregabalin
QOL: quality of life
RCT: randomized controlled trial
RoB: risk of bias
RR: risk ratio
SD: standard deviation
SMD: standardized mean difference
SOF: summary of findings
TAP: tapentadol
VAS: visual analogue scale
