# Supplementary material for: Benefits and safety of gabapentinoids in chronic low back pain: A systematic review and meta-analysis of randomized controlled trials
Source: PLoS Med. 2017 Aug 15;14(8):e1002369. doi: 10.1371/journal.pmed.1002369 (PMC5557428; doi:10.1371/journal.pmed.1002369)
Supplement: S1 Text — (DOCX) [file pmed.1002369.s001.docx]

**S1 Text: Search Strategy for MEDLINE and EMBASE**

Database: Ovid MEDLINE(R) In-Process & Other Non-Indexed Citations, Ovid MEDLINE(R) Daily and Ovid MEDLINE(R) <1946 to Present>

Search Strategy:

1 exp Back Pain/ (32247)

2 low back pain.mp. (26237)

3 dorsalgia.mp. (75)

4 back ache.mp. (85)

5 (lumbar adj pain).mp. [mp=title, abstract, original title, name of substance word, subject heading word, keyword heading word, protocol supplementary concept word, rare disease supplementary concept word, unique identifier] (1238)

6 exp Coccyx/ or coccydynia.mp. (970)

7 exp Spondylosis/ (6155)

8 lumbago.mp. (1226)

9 back disorder.mp. (116)

10 1 or 2 or 3 or 4 or 5 or 6 or 7 or 8 or 9 (46531)

11 exp Anticonvulsants/ or exp gamma-Aminobutyric Acid/ or gabapentin.mp. (162086)

12 gaba agents.mp. or exp GABA Agents/ (147096)

13 gabapentinoids.mp. (95)

14 pregabalin.mp. or exp Pregabalin/ (2385)

15 lyrica.mp. (88)

16 neurontin.mp. (144)

17 11 or 12 or 13 or 14 or 15 or 16 (212270)

18 10 and 17 (211)

Database: Embase <1974 to 2017 May 02>

Search Strategy:

--------------------------------------------------------------------------------

1 exp backache/ (86852)

2 backache.mp. (44068)

3 exp Low Back Pain/ (45830)

4 low back pain.mp. or low back pain/ (51243)

5 lumbago.mp. (1637)

6 spondylosis.mp. (8248)

7 or/1-6 (99535)

8 gabapentin.mp. or exp gabapentin/ (25534)

9 anticonvulsants.mp. or exp anticonvulsive agent/ (360788)

10 neurontin.mp. (1950)

11 gabapentinoids.mp. (202)

12 pregabalin/ (10346)

13 pregabalin.mp. (10607)

14 lyrica.mp. (942)

15 or/8-14 (361243)

16 7 and 15 (3035)

17 limit 16 to english language (2864)

18 16 not 17 (171)
