# Supplementary material for: Benefits and safety of gabapentinoids in chronic low back pain: A systematic review and meta-analysis of randomized controlled trials
Source: PLoS Med. 2017 Aug 15;14(8):e1002369. doi: 10.1371/journal.pmed.1002369 (PMC5557428; doi:10.1371/journal.pmed.1002369)

**Supplementary file 3: Rescaling or Conversion of Pain Scores to a Common 0-10 Numerical Rating Scale.**

**Conversion to natural units of most familiar/used or reference instrument [0-10 NRS]**

- Instrument A (reference instrument of 0-10 NRS); Scale: L_A_ and U_A_; Range: *R = R_A_ = U_A_–L_A_*
- Instrument B (another instrument) used in Trial *i*: Scale: L_B_ and U_B_; Range: *R = R_B_ = U_B_–L_B_*
- *C*: control group; *m^B^Ci* and *sd^B^Ci* : mean and sd of control group
- *E*: experimental group; *m^B^Ei* and *sd^B^Ei* : mean and sd of intervention group

We need to obtain estimates of, *m^A^Ci , sd^A^Ci , m^A^Ei* , and *sd^A^Ei* , of what would have been observed had instrument A been used in trial *i*


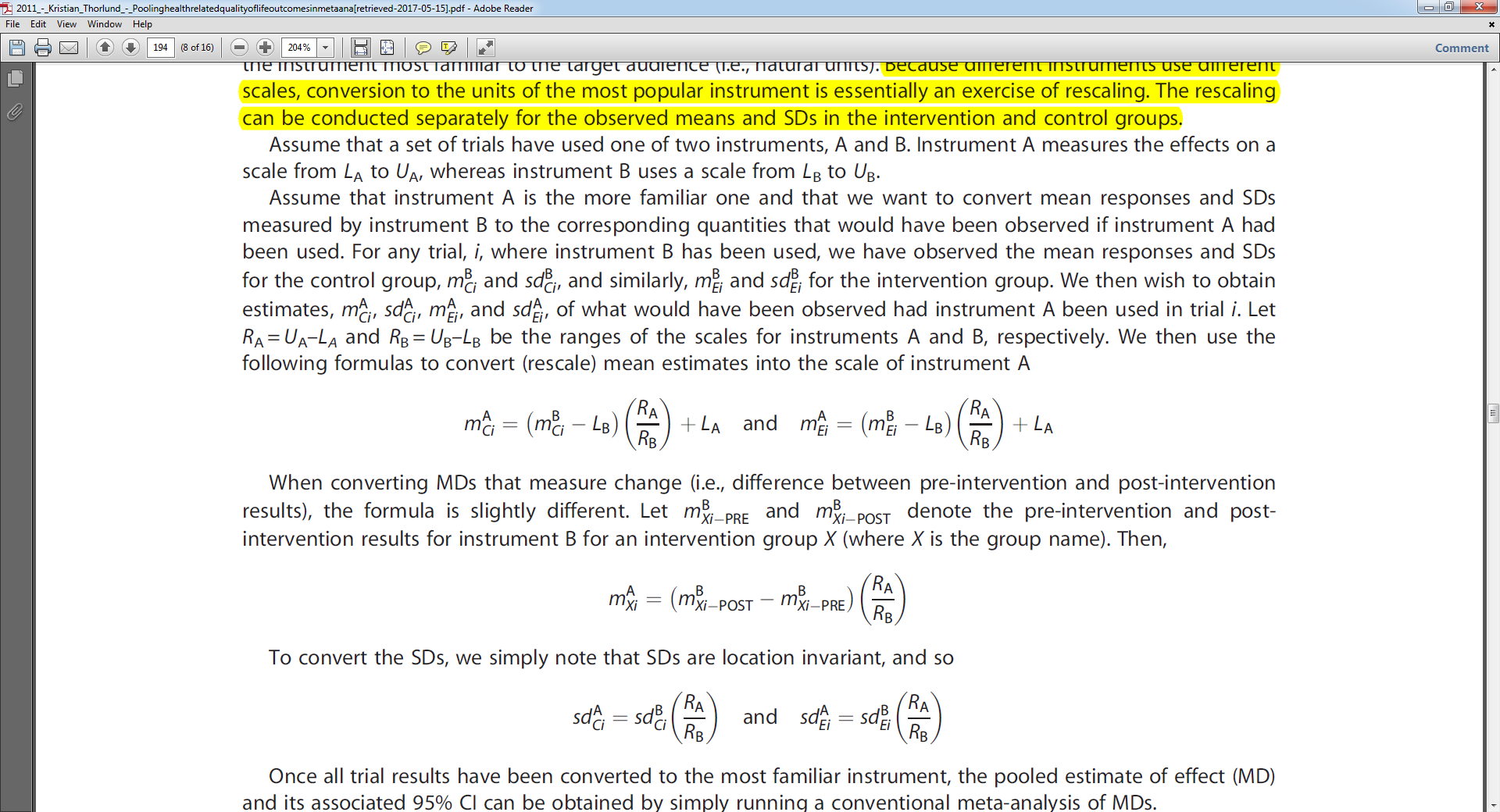


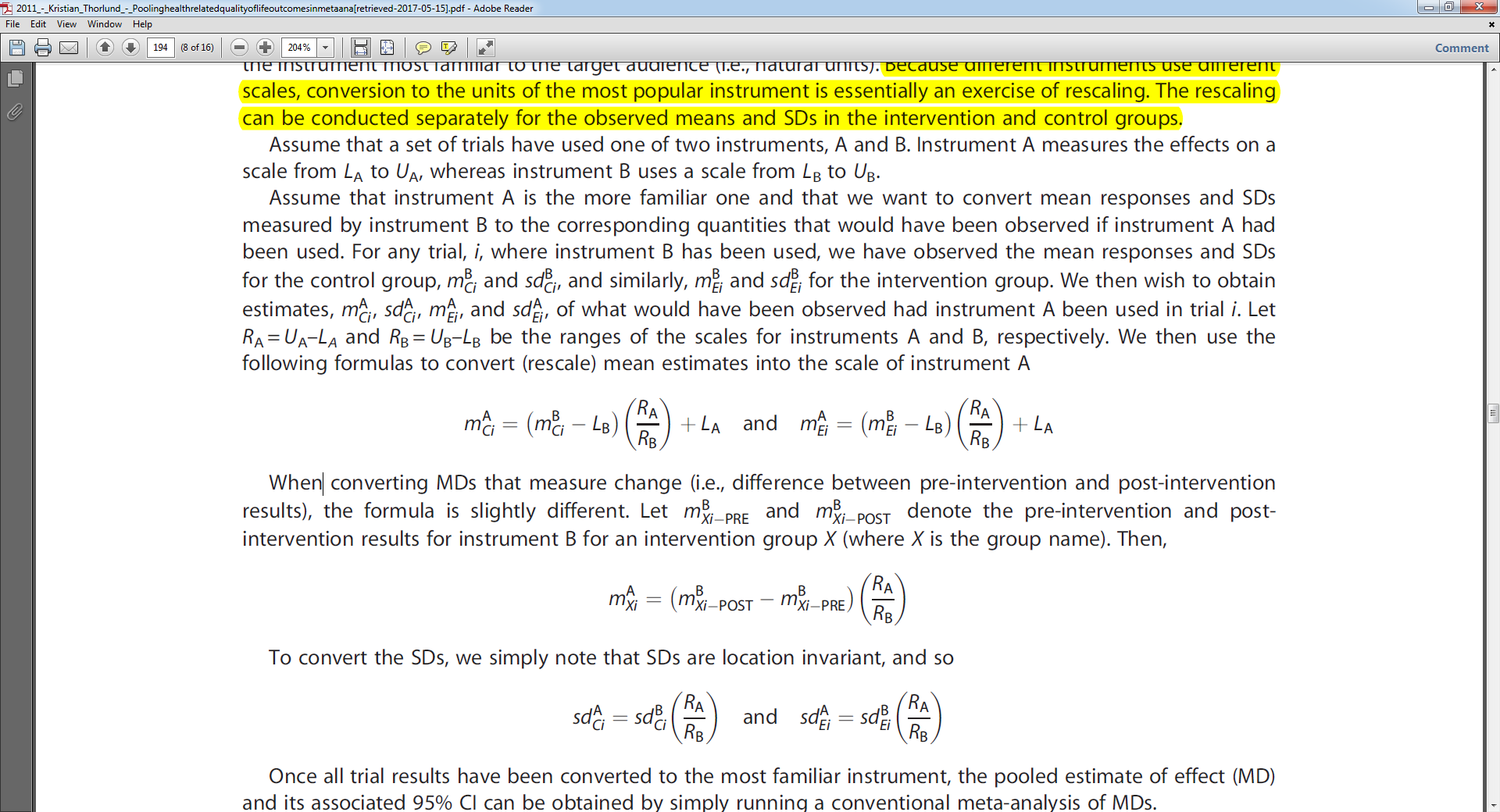

Supplement: S2 Text — (DOCX) [file pmed.1002369.s002.docx]
