# Supplementary figures and images for: Benefits and safety of gabapentinoids in chronic low back pain: A systematic review and meta-analysis of randomized controlled trials
Source: PLoS Med. 2017 Aug 15;14(8):e1002369. doi: 10.1371/journal.pmed.1002369 (PMC5557428; doi:10.1371/journal.pmed.1002369)

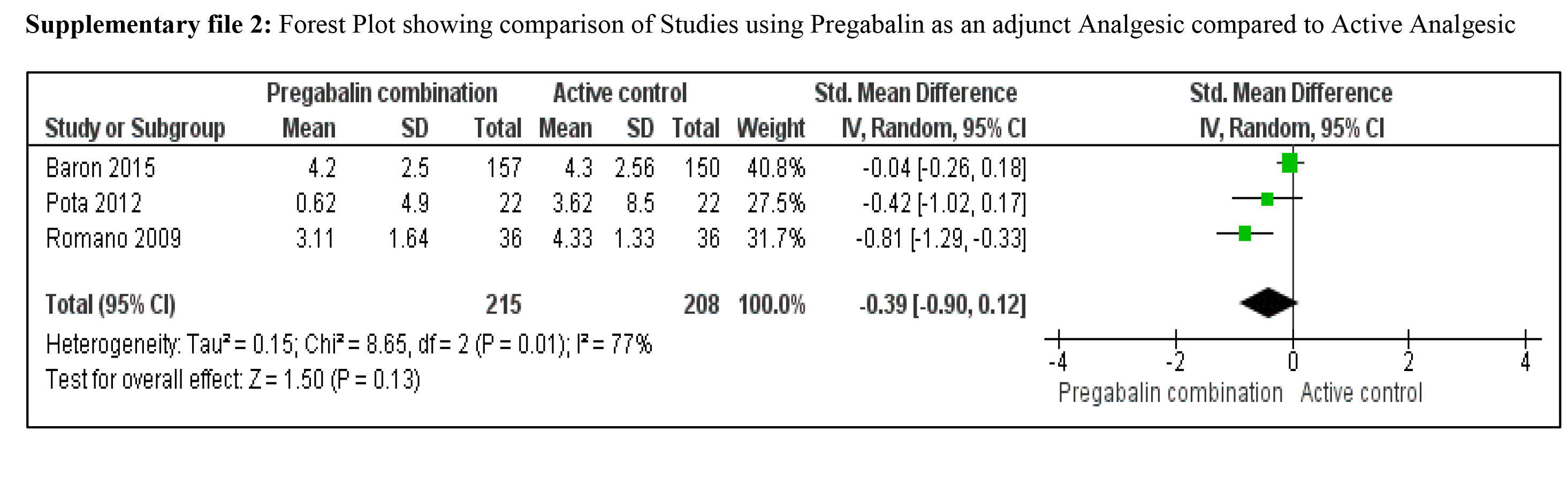

Supplement: S1 Fig — (TIF) [file pmed.1002369.s003.tif]
